# Effects of* Juglans regia* Root Bark Extract on Platelet Aggregation, Bleeding Time, and Plasmatic Coagulation:* In Vitro* and* Ex Vivo* Experiments

**DOI:** 10.1155/2018/7313517

**Published:** 2018-08-14

**Authors:** A. Amirou, M. Bnouham, A. Legssyer, A. Ziyyat, M. Aziz, M. Berrabah, H. Mekhfi

**Affiliations:** ^1^Laboratory of Physiology, Genetic and Ethnopharmacology, Faculty of Sciences, Mohammed the First University, Oujda, Morocco; ^2^Laboratory of Mineral Solid and Analytical Chemistry, Department of Chemistry, Faculty of Sciences, Mohammed the First University, Oujda, Morocco

## Abstract

Platelets have an important role in thrombosis and haemostasis. Hyperactivity of the platelets has been associated with thromboembolic diseases and represents the main cause of complications of cardiovascular diseases. Crude aqueous extract (CAE) of* Juglans regia *root bark was evaluated for bleeding time, antiaggregant activity by using agonists, thrombin, ADP, collagen, or arachidonic acid (*in vitro* and* ex vivo*), and anticoagulant activity by measuring the clotting parameters: activated partial thromboplastin time, prothrombin time, thrombin time, and fibrinogen dosage (*in vitro* and* ex vivo*). The result of this study reported that the strongest antiaggregant effect of CAE* in vitro* was observed on the ADP-induced aggregation with inhibitions up to 90 %, while, in ex vivo experiments, the inhibition (more than 80 %) was observed with all agonists. Anticoagulant effect of CAE significantly prolonged the TT and decreased the fibrinogen level* in vitro* and* ex vivo* without interfering with APTT and PT. The bleeding time in mice and rats was significantly increased by CAE. The antiplatelet and anticoagulant effect observed in this study suggest that* Juglans regia* could have antithrombotic and/or thrombolytic activities and provide an alternative therapy against thrombotic complications related to cardiovascular diseases.

## 1. Introduction 

Thrombosis is the formation of blood clots (thrombus) in the circulatory system caused by the imbalance of homeostatic system. The majority of cardiovascular diseases, including acute coronary syndrome, venous thromboembolism, deep vein thrombosis, pulmonary embolism, myocardial infarction, atherosclerosis, and ischemic stroke, are associated with thrombotic disorders, resulting in serious consequences such as sudden death [1, 2].

The therapies with antithrombotic, anticoagulant, and antiaggregant drugs are still widely used for prevention or for treatment of thrombotic diseases, despite side effects like gastrointestinal symptoms and hemorrhage [3]. Nowadays, scientist carried so much interest toward the development of new antithrombotic agents from the natural products, especially when a lot of people around the world use plants as a remedy against various diseases [4, 5]. Many plants have been investigated in our laboratory for their antiaggregant potential such as* Petroselinum crispum *[6] and* Argania spinosa *[7]. Jin et al. [8] reported that several antithrombotic agents have been explored like* Thymus vulgaris, Rosmarinus officinalis, Lavandula hybrida, Allium cepa, Ginkgo biloba*, and* Olea europaea*.

*Juglans regia (Jr) *or walnut (family of Juglandaceae) is commonly known in Morocco as “Gargae” or “Jawz” for the fruit and “Souak” for the root bark. All parts (bark, leaves, flowers, seed, and fruit) have been widely used in folk medicine to treat many health disorders. Bnouham et al. [9] reported in their reviews that the fruit of* Jr* is used traditionally in Morocco as hypoglycemic, anthelmintic, antiseptic, tonic, and astringent, and the leaves and cortex were indicated as depurative, cicatrizing, stomach disorders, and antidote poison. Also, Merzouki et al. [10], in their survey, mentioned that the root bark was prescribed for tooth care and gingivitis.

Experimentally, biological activities of* Jr* have been reported in several reviews, such antioxidant and antitumor, anti-inflammatory, anticarcinogenic, antihyperglycemic activities, and cardioprotective properties [11, 12]. Also, Nergiz-Ünal et al. [13] reported that the dietary intake of walnut reduces the atherosclerotic plaque formation in ApoE-deficient mice. In addition to that, Rywaniak et al. [14] mentioned that extract of* Jr* husks significantly reduced ADP-induced platelet aggregation in whole blood.

However, no study has yet been performed on the antiplatelet and anticoagulant activities of* Jr *root bark. The aim of the present study was to investigate the effect of CAE of* Jr* root bark on platelet aggregation, coagulation, and bleeding time, in* in vitro* and in* ex vivo *experiments.

## 2. Materials and Methods

### 2.1. Plant Collection

The root bark of walnut (*Juglans regia L*, Juglandaceae) was collected in January 2014, M'semrir (province of Tinghir in the Southern Morocco). The plant was identified by Professor Mohammed Fennane, an expert botanist from the Scientific National Institute, Rabat (Morocco), and a voucher specimen (HUMPO 149) was deposited in the herbarium at University Mohammed the First (Oujda, Morocco). The bark of* Jr* was washed with water and dried with air in dark place. The dried bark was powdered by Moulinex Blender and kept in a dark until time of use.

### 2.2. Crude Aqueous Extract Preparation

The CAE was prepared by infusion of dried bark of* Jr* (25 g) with boiling distilled water (500 ml) for 30 minutes. The infusate was filtered and evaporated by rotary evaporation (Heidolph Instruments, Germany) at a temperature of 45°C. The yield of extraction was 11.8 %.

### 2.3. Experimental Animals

Wistar rats and albino mice were housed in an animal unit (Faculty of Sciences, Oujda, Morocco). They were maintained under standard laboratory conditions (12 h light/dark cycle, temperature 22 ± 2°C, and with free access of food and water). Experimental protocols were in compliance with the Guide for the Care and Use of Laboratory Animals of the US Department of Health and Human Services (NIH publication no 85–23, revised 1985).

### 2.4. *In Vitro* Antiplatelet Activity

#### 2.4.1. Washed Platelets Preparation

Washed platelets (WP) were prepared as described by Mekhfi et al. [7]. Animals were slighting anesthetized with ether. Blood was taken by catheterization from the abdominal aorta and deposited into a plastic tube containing anticoagulant solution (9:1, v/v) (citric acid 130 mM, trisodium citrate 170 mM, and dextrose 4%). The blood was immediately centrifuged at 230 g for 15 min to separate the plasma rich platelet (PRP). PRP was recuperated and centrifuged for 8 min at 120 g to eliminate residual blood cells and for 15 min at 400 g to obtain the platelet pellet. Then, the platelet pellet was suspended in the wash buffer (NaCl 137 mM, KCl 2.6 mM, NaHCO_3_ 12 mM, MgCl_2_ 0.9 mM, Glucose 5.5 mM, CaCl_2_ 1.3 mM, Gelatin 0.25%, and pH 6.5) and centrifuged at 400 g for 15 min. Finally, the platelets were resuspended in an adequate volume of the final buffer (NaCl 137 mM, KCl 2.6 mM, MgCl_2_ 0.9 mM, Glucose 5.5 mM, CaCl_2_ 1.3 mM, Gelatin 0.25%, Hepes 5 mM, and pH 7.4) to make a WP suspension with approximately 5×10^5^ cells/mm^3^. The prepared WP were used immediately in the same day.

#### 2.4.2. Platelet Aggregation Study

Platelet aggregation studies were performed using an aggregometer (Chrono-Log, Aggregometer, Kordia, USA). Aggregation was measured by the change in light transmission, with the value for the blank sample (buffer without platelets) set at 100%. For the control test, an aliquot of WP (250 *μ*l) was incubated at 37°C with 1000 rpm. After the incubation period, platelet aggregation was induced by the addition of either final concentration: thrombin 0.5 U/ml, ADP 5 *μ*M, and collagen 5 *μ*g/ml. Aggregation signal was recorded for 5 min on a paper recorder (Leybold-Heraeus, Austria). For plant tests, WP (250 *μ*l) were preincubated with the CAE of* Jr *(1 mg/ml) for 1 min at 37°C and then activated by agonists.

The parameters measured are as follows:

(i) The amount of aggregation (%).
(ii) The inhibition of platelet aggregation (Y, %) calculated using the following equation:
  Y (%) = [(A−B) /A] ×100,

where A is the maximum aggregation of the control and B is the maximum aggregation with plant extract.

### 2.5. Bleeding Time Assay

Albino mice male and female (18-22 g) were randomly divided into four groups of 5 animals per group, treated orally as follows: control group received distilled water (1 ml/100g), groups of CAE received* Jr *(1 and 1.5 g/kg), and the last group received acetyl salicylic acid (ASA) (30 mg/Kg/day). One hour after a single administration, animals were anaesthetised intraperitoneally by sodium pentobarbital (50 mg/kg) and placed on a hotplate. Bleeding was assessed by amputating 1.5 cm of the tail tip with a scalpel and blood was blotted into a filter paper. The time between amputation and bleeding cessation was recorded as the bleeding time (BT).

### 2.6. *In Vitro* Anticoagulant Activity

#### 2.6.1. Preparation of Plasma Sample

This study was realized on plasmatic samples. After anesthesia of the rats (250–300 g) with ether, blood was collected from the abdominal aorta directly into citrated tubes (trisodium citrate 3.8%, 1/9; v/v). Blood was immediately centrifuged at 3000 rpm for 20 min, to separate the blood cells from platelet poor plasma (PPP). The PPP was collected and deposited in a plastic tube.

#### 2.6.2. Experimental Protocols

Plasma coagulation experiments were performed by using a semiautomatic coagulometer (Thrombostat, Behnk Elektronik, Norderstedt, Germany).

Plasma mixtures (100 *μ*l of the PPP with 50 *μ*l of different concentrations of CAE of* Jr *(0.25, 0.5, and 1 mg/ml) or 50 *μ*l of distilled water) were incubated at 37°C for 5 min before performing activated partial thromboplastin time (APTT), prothrombin time (PT), and thrombin time (TT) assays. For the fibrinogen assay, PPP was diluted with a suitable buffer. An aliquot of 100 *μ*l of this PPP was incubated, for 300 s, with 100 *μ*l of CAE of Jr at 37°C, and then the time of clot formation was measured. Determination of clotting times (APTT, PT, and TT) and fibrinogen level were carried out in accordance with the manufacturer's recommended protocols. Heparin (0.4 U/ml) was used as positive control for coagulation test.

### 2.7. *Ex Vivo* Experiments

Wistar rats (120 - 210 g) were subjected to subchronic treatment. Animals were divided randomly into four groups:

1. Control group receiving distilled water (1 ml/100 g /day).
2. Test group receiving the CAE of* Jr* (250 mg/Kg/day).
3. Positive control group receiving ASA (30 mg/Kg/day) for tail bleeding time and aggregation tests.
4. Positive control group receiving warfarin (1 mg/Kg) for coagulation test (a single administration).

All doses were administered orally every morning for 30 days.

#### 2.7.1. Bleeding Time Assay

One hour after the last administration, animals were anesthetized by intraperitoneal injection of sodium pentobarbital (50 mg/Kg) and placed on a hotplate at 37°C. Bleeding was assessed by amputating 5 mm of the tail tip and the duration between tail incision and bleeding stops is the bleeding time (s).

After BT determination, the blood was collected into two tubes, used for aggregation and coagulation.

#### 2.7.2. Platelet Aggregation Study

Whole blood was collected, WP were prepared, and platelet aggregation was performed as described above.

#### 2.7.3. Plasmatic Coagulation Assay

PPP, obtained after centrifugation, was used for the clotting times (APTT, PT, and TT) and fibrinogen determination. These measurements were performed for each group (control and treated), using a commercial diagnostics kits by following the manufacturers' instructions.

*Reagents. *ADP was purchased from Verum Diagnostica GmbH (Munich, Germany), collagen calf skin type III from Sigma (USA), thrombin from Sigma (Germany), arachidonic acid from CALBIOCHEM (USA) or Cayman Chemical Company (USA), and warfarin from Sigma (Canada).

*Statistical Analysis. *Statistical analysis data were performed by using GraphPad Prism Software version 5.01 (GraphPad Software, Inc.). Statistical significance between two groups was determined using Student's t-test. For the treatment studies, the analysis of variance (ANOVA) followed by the Dunnett's test was used. All results are expressed mean ± SEM and “p” values lower than 0.05 were considered statistically significant.

## 3. Result

### 3.1. Antiaggregant Effect of Aqueous* Juglans regia* Bark Extract

**Figure 1:**
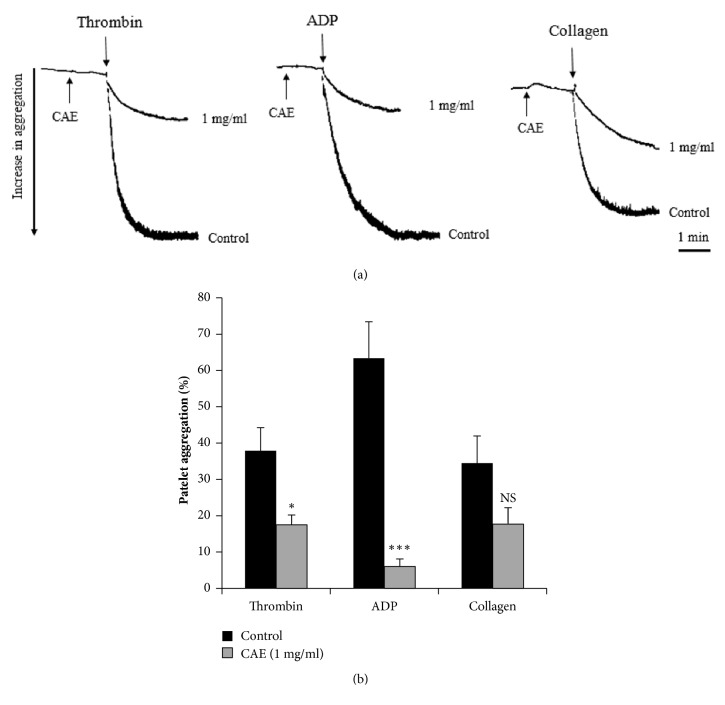
Effect of* Juglans regia* CAE (1 mg/ml) on platelet aggregation induced* in vitro* by thrombin (0.5 U/ml), ADP (5 *μ*M) or collagen (5 *μ*g/ml). ^*∗*^ p < 0.05; ^*∗∗∗*^ p < 0.001 compared to control; NS: not significant; n = 5. (a) Original tracing and (b) histograms showed the aggregation platelet.

After preincubation with the CAE of* Jr *bark for 1 min, platelets were stimulated by ADP (5 *μ*M), thrombin (0.5 U/ml), or collagen (5 *μ*g/ml).  Figure 1 showed that the CAE (1 mg/ml) inhibits platelet aggregation induced* in vitro* by ADP, thrombin, or collagen. The percentage of inhibition was 92 ± 3%, 51.14 ± 4.19%, and 44.45 ± 8%, respectively (n = 5). Regarding agonists, the strong inhibition of aggregation (p < 0.001) was observed with ADP then thrombin (p< 0.05) and collagen (no significant with p> 0.05).

### 3.2. Effect of* Juglans regia* CAE on Tail Bleeding Time

**Figure 2:**
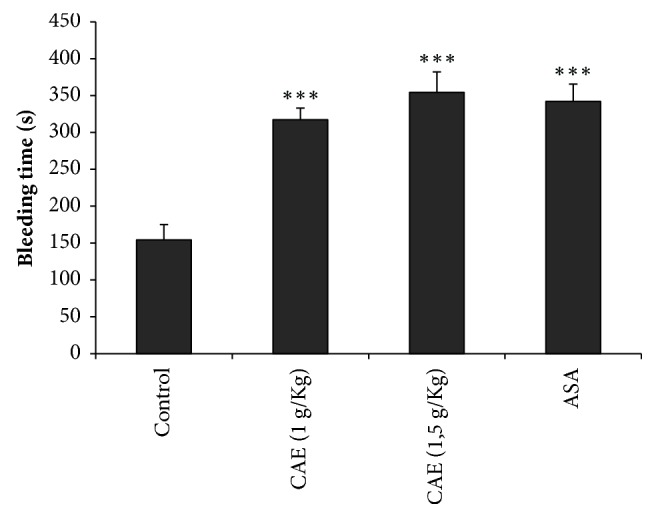
Effect of CAE on tail bleeding time in mice;^  *∗∗∗*^ p < 0.001 compared to control; n=5.

The BT was measured* in vivo,* one hour after the oral administration of distilled water, CAE of* Jr*, and ASA. As shown in Figure 2, the two doses of* Jr *bark, 1 and 1.5 g/Kg, significantly (p < 0.001) prolonged the BT (317.3 ± 27.7 s and 354.5 ± 23.5 s, respectively), compared to the control group (154.2 ± 20.8 s). Also, the ASA significantly (p < 0.001) prolonged the BT (342.1 ± 23.5 s).

### 3.3. Anticoagulant Effect of CAE of* Juglans regia* Bark

Anticoagulant effect of CAE (0.25, 0.5, and 1 mg/ml) was explored by evaluating coagulation times (PT, APTT, and TT) and fibrinogen concentration in PPP from rats. As shown in Table 1, the CAE of* Jr* at 0.25 and 0.5 mg/ml prolonged significantly the TT (p<0.05 and p<0.01, respectively) and reduced the fibrinogen concentration (p<0.001) when compared to the control. The PT and APTT still unchanged (p>0.05), while, at 1 mg/ml of CAE, all clotting times, PT (p< 0.05), APTT (p<0.01), and TT (p<0.001), were extended and the plasmatic fibrinogen level was reduced (p<0.001). As expected, heparin (0.4 U/ml), used as anticoagulant drug reference, affects (p<0.001) in the same manner all these parameters.

### 3.4. *Ex Vivo* Experiments

#### 3.4.1. Effect of CAE of* Jr* on* Ex Vivo* Bleeding Time

The BT of rats was measured to determine the effect of CAE of* Jr* bark (250 mg/Kg/day) on platelet function after 4 weeks of treatment. The result shows that BT was significantly prolonged (n = 5) by CAE of* Jr* (304.32 ± 24.72 s, p < 0.01) as well as ASA (327.12 ± 9.914 s, p < 0.001) when compared to control (194.4 ± 5.87 s).

#### 3.4.2. Antiaggregant Effect of the CAE of* Jr Ex Vivo*

**Figure 3:**
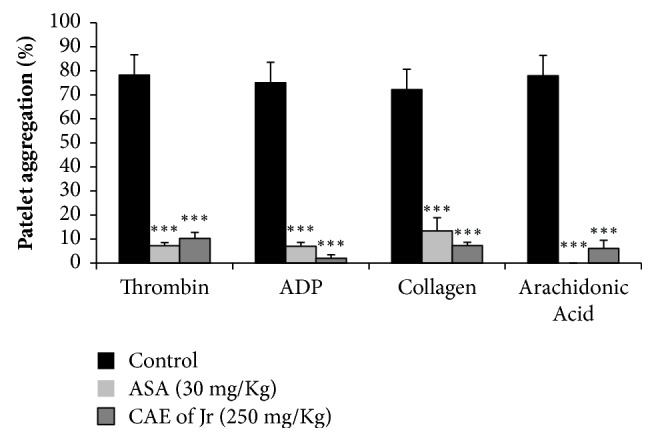
Effect of CAE of* Jr* and ASA on* ex vivo* platelet aggregation induced by thrombin (0.5 U/ml), ADP (5 *μ*M), collagen (5 *μ*g/ml), and arachidonic acid (20 *μ*M). Mean ± SEM; ^*∗∗∗*^p < 0.001; n =5.

The CAE of* Jr *effect (250 mg/Kg/day) was evaluated on rat platelet aggregation after 4 weeks of treatments.  Figure 3 shows that the extract as well as ASA (30 mg/Kg/day) reduced significantly (p < 0.001) the amount of platelet aggregation induced by thrombin, ADP, collagen, or arachidonic acid compared to the control group. In all cases, the inhibition of aggregation was more than 85%.

#### 3.4.3. *Ex Vivo* Anticoagulant Effect of CAE of* Jr*

The APTT, PT, TT, and fibrinogen concentration were measured in PPP to assess the* ex vivo* anticoagulant activity of CAE. As shown in Table 2, the bark of* Jr* significantly prolonged the TT (p < 0.01) and decreased the fibrinogen level (p < 0.001), but still without any significant effect on PT and APTT when compared to the control group. Warfarin, used as an anticoagulant drug in the prevention of blood clots formation, significantly (p < 0.001) prolonged all the clotting times and decreased fibrinogen level.

## 4. Discussion

Platelets play a critical role in hemostasis and their major purpose is to plug the injuries in the vessel walls. They are also an important contributor in the development of abnormal thrombosis that is related to the cardiovascular diseases [15]. Despite the existence of a well know antithrombotic remedies with a proven efficacy, the research of novel bioactive natural products interfering with platelets and/or plasmatic coagulation is increasingly intense. The optimal bioactive compound may be efficient but with less side effects like bleeding.

In this study, we presented crude aqueous extract of* Juglans regia* root bark as a potent extract that affects primary and secondary haemostasis. The primary haemostasis is commonly explored by platelet aggregation and bleeding time measurements, while the secondary haemostasis is investigated by plasmatic coagulation times (PT, APTT, and TT) and fibrinogen determinations. In the* in vitro* experiments, the obtained data show that CAE of* Jr *at 1 mg/ml exhibits a strong inhibition of platelet aggregation induced by ADP, thrombin, not by collagen, and a significant prolongation of BT. In practice, BT is a useful test to detect abnormal platelet function. Its extension in our study confirms the antiaggregant activity of* Jr* extract. Scientific literature reported that several plants have shown inhibition of platelet aggregation with or without affecting BT such as* Kyung-Ok-Ko* (mixture of several medicinal plants) [16],* Lagenaria siceraria *[17],* argan oil* [7],* Asarum sieboldii* [18],* Geoffroea spinosa *[19], and* Mucuna pruriens *[20]. In general, speculations about molecular mechanism explaining the antiaggregant activity evoke many signaling pathways, including those mediated by phospholipase C (PLC) [21], phospholipase A2 (PLA2) and thromboxane A2 (TXA2), or the stimulation of adenylate cyclase (cAMP) and guanylate cyclase (cGMP) [22]. In* ex vivo* results, the antiaggregant effect of* Jr* extract was observed with all used platelets activators: ADP, thrombin, collagen, and arachidonic acid. It is well known that each agonist attaches to its specific platelet receptor, then mobilizes intracellular messengers (cAMP, cGMP), and activates enzymes. Bioactive compounds, present in* Jr* extract, may probably interfere negatively with ADP (P2Y1, P2Y12), thrombin (PAR 1 and PAR 2), and collagen or arachidonic receptors. Theses interactions cause finally a fall in intracellular calcium. In the coagulation cascade (intrinsic, extrinsic, and common phases), parameters such as PT, APTT, TT, and fibrinogen concentration are the basic blood tests for evaluating hemorrhage and thrombosis risk. APTT is related to the intrinsic and/or common pathways of plasmatic coagulation and is used to detect deficiencies of factors II, V, VIII, IX, X, XI, and XII. PT explores the extrinsic phase for detecting bleeding disorders and affected clothing factors in the extrinsic or common pathways. TT and fibrinogen amount concern the common and ultimate coagulation phase and their determinations explore the ability of thrombin to catalyze the polymerization of fibrinogen to form fibrin [23, 24]. Our experiments demonstrate that CAE of* Jr*, at lower concentrations, significantly extends TT and diminishes fibrinogen content. But, at 1 mg/ml, all coagulation parameters are modified in the same way. These observations let us to suggest that CAE of plant exerts a strong action on the common pathway, more than the extrinsic and intrinsic pathways of plasmatic coagulation. In this context, the use of heparin as an anticoagulant and antithrombotic drug in therapeutic [25] confirms and validates the* Jr *CAE results. In order to verify if CAE of* Jr* is still efficient* in vivo*, animals were fed with plant extract for one month and, at the end of treatment, all parameters were performed in* ex vivo*. The obtained results showed that, in treated rats, CAE exerts significantly the same effects comparing to* in vitro *studies, suppression of platelet aggregation, prolongations of BT and TT, and reduction of plasmatic fibrinogen level, while APTT and TP remain unchanged in* ex vivo* conditions.

The main objective to use the CAE in our investigation is to confirm the medicinal properties of this plant, as possible as, in the same conditions of population use. With this type of extract, it is not easy to relate exactly the observed effects to one or more compounds because the CAE constitute a complex and important mixture of bioactive substances that may probably act in synergy.

Phytochemical analysis of methanolic and aqueous extracts of* Jr* bark revealed the presence of many and diverse compound families [26, 27], among them, polyphenols, which constitute a large group of natural products widely found in different vegetables. This class is divided into several subclasses such as phenolic acids, flavonoids, lignans, and tannins. These compounds are well known by their broad range of cardiovascular activities like vasodilating, antiplatelet aggregation [28]. In their review, Middleton et al. [29] reported several studies related the beneficial effect of flavonoids in hemostasis and thrombosis. These authors mentioned that flavonoids significantly inhibited platelet function (adhesion, aggregation, and secretion). In this domain, the mechanism of action of flavonoids is well documented. Some flavonoid molecules like flavone, chrysin, apigenin, and phloretin act by depressing platelet cyclooxygenase (CO) activity and decreased the cAMP response to prostacyclin [29]. Recently, Du et al. [30] reported that quercetin and rutin are the most flavonoids commonly studied for their cardiovascular effects. Quercetin was an effective inhibitor of lipoxygenase (12-LO) activity in human platelets and its action has been related to the inhibition of arachidonic acid metabolism by CO [29]. Choi et al. [31] demonstrated that rutin inhibited collagen induced platelet aggregation in human platelets and prolonged APTT and PT. Furthermore, Correia-Da-Silva et al. [32] reported that transresveratrol 3-ß-D-glucopyranoside persulfate and sulfated oligoflavonoids have been described as anticoagulant/antiplatelet agents.

The involvement of such compounds in hemostasis was already initiated in our laboratory. Flavonoids (genins and heterosidic flavonoids) isolated from* Arbutus unedo* [33] or* Petroselinum crispum *[6] inhibit platelet aggregation. More, Gadi et al. [6] reported that the adhesion of human platelets to collagen under flow was greatly decreased by genins of* Petroselinum crispum*.

## 5. Conclusion

In the present study, the crude aqueous extract of* Juglans regia* root bark exerts* in vitro* and* ex vivo* potent antiplatelet and anticoagulant effects. These data suggest that* Juglans regia* could be a promising therapy preventing thrombotic complications of cardiovascular disease.

## Figures and Tables

**Table 1:** Effect of the CAE of *Juglans regia* on APTT, PT, and TT and fibrinogen concentration.

**Sample**	**Doses**	**PT (s)**	**APTT (s)**	**TT (s)**	**Fibrinogen (g/l)**
**Control**	(1:1/v,v)	16.62 ± 1.16	13.96 ± 0.57	43.29 ± 2.20	1.17 ± 0.09

***Juglans regia* (mg/ml)**	0.25	20.12 ± 1.13 ^NS^	19.74 ± 1.27 ^NS^	69.5 ± 1.67 ^*∗*^	0.68 ± 0.05 ^*∗∗∗*^
	0.5	19.62 ± 1.53 ^NS^	19.18 ± 1.55 ^NS^	80.24 ± 3.21 ^*∗∗*^	0.64 ± 0.04 ^*∗∗∗*^
	1	20.55 ± 0.76 ^*∗*^	25.06 ± 3.54 ^*∗∗*^	190.8 ± 11.63 ^*∗∗∗*^	0.49 ± 0.02 ^*∗∗∗*^

**Heparin (U/ml)**	0.4	53.48 ± 4.4^*∗∗∗*^	>300	>300	0.53 ± 0.03 ^*∗∗∗*^

Mean ± SEM, PT: prothrombin time, APTT: activated partial thromboplastin time, TT: thrombin time, ^*∗*^   p <0.05, ^*∗∗*^  p <0.01, ^*∗∗∗*^  p<0.001, and NS: no significant. n =5

**Table 2:** Effect of CAE of *Jr* on *ex vivo* on PT, APTT, and TT and fibrinogen concentration.

**Sample**	**Doses**	**PT (s)**	**APTT (s)**	**TT (s)**	**Fibrinogen (g/l)**
**Distilled water (ml/100g)**	1	18.26 ± 1.51	16.40 ± 1.46	39.62 ± 7.39	2.58 ± 0.07

**CAE of *Jr* (mg/Kg)**	250	20.94 ± 0.39 ^NS^	17.48 ± 1.81 ^NS^	54.96 ± 3.46 ^*∗∗*^	1.98 ± 0.10 ^*∗∗∗*^

**Warfarin (mg/Kg)**	1	33.30 ± 3.44 ^*∗∗*^	36.94 ± 3.06 ^*∗∗∗*^	117.96± 10.27 ^*∗∗∗*^	1.55 ± 0.06 ^*∗∗∗*^

Mean ± SEM, ^*∗∗*^ p < 0.01, ^*∗∗∗*^ p < 0.001, NS: not significant, and n = 5.

## Data Availability

All data used to support this study are available to the reader in the Laboratory of Physiology, Genetics and Ethnopharmacology, Faculty of Sciences, Université Mohammed Premier, Oujda, Morocco.
